# Differential Recognition of *P. falciparum* VAR2CSA Domains by Naturally Acquired Antibodies in Pregnant Women from a Malaria Endemic Area

**DOI:** 10.1371/journal.pone.0009230

**Published:** 2010-02-16

**Authors:** Kim J. M. Brolin, Kristina E. M. Persson, Mats Wahlgren, Stephen J. Rogerson, Qijun Chen

**Affiliations:** 1 Department of Microbiology, Tumor and Cell Biology, Karolinska Institutet, Stockholm, Sweden; 2 Department of Parasitology, Swedish Institute for Infectious Disease Control (SMI), Stockholm, Sweden; 3 Department of Medicine (RMH/WH), The University of Melbourne, Melbourne, Victoria, Australia; 4 Key Laboratory of Zoonosis, Ministry of Education and Jilin University, Changchun, China; 5 Institute of Pathogen Biology, Chinese Academy of Medical Science, Beijing, China; World Health Organisation, Switzerland

## Abstract

**Background:**

*Plasmodium falciparum* infected red blood cells (iRBC) express variant surface antigens (VSA) of which VAR2CSA is involved in placental sequestration and causes pregnancy-associated malaria (PAM). Primigravidae are most susceptible to PAM whereas antibodies associated with protection are often present at higher levels in multigravid women. However, HIV co-infection with malaria has been shown to alter this parity-dependent acquisition of immunity, with more severe symptoms as well as more malaria episodes in HIV positive women versus HIV negative women of a similar parity.

**Methods:**

Using VAR2CSA DBL-domains expressed on the surface of CHO-745 cells we quantified levels of DBL-domain specific IgG in sera from pregnant Malawian women by flow cytometry. Dissociations constants of DBL5ε specific antibodies were determined using a surface plasmon resonance technique, as an indication of antibody affinities.

**Results:**

VAR2CSA DBL5ε was recognized in a gender and parity-dependent manner with anti-DBL5ε IgG correlating significantly with IgG levels to VSA-PAM on the iRBC surface. HIV positive women had lower levels of anti-DBL5ε IgG than HIV negative women of similar parity. In primigravidae, antibodies in HIV positive women also showed significantly lower affinity to VAR2CSA DBL5ε.

**Conclusions:**

Pregnant women from a malaria-endemic area had increased levels of anti-DBL5ε IgG by parity, indicating this domain of VAR2CSA to be a promising vaccine candidate against PAM. However, it is important to consider co-infection with HIV, as this seems to change the properties of antibody response against malaria. Understanding the characteristics of antibody response against VAR2CSA is undoubtedly imperative in order to design a functional and efficient vaccine against PAM.

## Introduction

Pregnancy-associated malaria (PAM) has a major impact on the mother and child [1] and is often associated with the sequestration of *Plasmodium falciparum* infected red blood cells (iRBC) in the placenta [2], [3]. IRBC expressing the adhesin VAR2CSA adhere to the receptor CSA expressed by syncytiotrophoblast [4], [5], [6], [7], and potentially enable the parasites to evade the immune system and successfully proliferate. Hence, despite previously acquired immunity to non-VAR2CSA expressing *P. falciparum* malaria, pregnant women are highly susceptible to PAM [8], leading to maternal anemia, low birth weight, miscarriage and stillbirths [9], [10], [11].

VAR2CSA is a member of the PfEMP1 family but displays a different domain architecture than typical PfEMP1s and has an unusual high sequence conservation between isolates [12], [13]. VAR2CSA contains six Duffy-binding-like (DBL) domains of which four (DBL2x, DBL3x, DBL5ε and DBL6ε) domains have been shown to bind CSA *in vitro* [14], [15], [16]. Studies have shown that placental parasites and CSA-selected parasites bind normal non-immune IgG and IgM [17], [18], [19] and also that VAR2CSA DBL domains harbour non-immune IgG and IgM binding regions [19]. It has also been shown that immune antibodies are targeting various epitopes within the DBL-domains [20]. Antibody immunity against PAM is acquired in a parity dependent manner, and important functions of these antibodies are the ability to block adhesion to placental receptors [21] and to facilitate opsonic uptake by phagocytes [22], [23], [24]. VAR2CSA is the current vaccine candidate for PAM, but in order to design a rational and protective vaccine based on this large protein, increased knowledge of anti-VAR2CSA antibody response characteristics is needed. Since malaria and HIV co-exist to an extremely high extent in sub-Saharan Africa, it is also important to consider the fact that HIV positive women infected with malaria have more febrile illnesses, more anemia, and more adverse birth outcomes than HIV negative women with malaria [25]. Several studies have suggested HIV to affect the immune memory mechanism, which is responsible for the parity dependent acquisition of immunity to PAM, thus rendering women of all parities highly susceptible to PAM [26], [27]. HIV infection in multigravid women seems to impair the ability to control malaria parasitemia, resulting in more frequent and higher parasite density than in HIV negative women of the same parity [25]. HIV positive women in their first pregnancy do not experience a significantly increased risk of malaria prevalence, but do retain significantly higher parasite density [28]. In several studies, it has been shown that HIV positive women receiving intermittent preventive treatment in pregnancy (IPTp) need additional doses of drugs in order to be protected [28], [29], [30], further reflecting the need for increased knowledge of antibody dynamics.

*In vitro* studies often focus on investigating the mere presence of antibodies against the variant surface antigens present on the surface of iRBC causing pregnancy associated malaria (VSA-PAM), with less knowledge on the potentially important affinity to their target. Being a neglected factor in PAM immune response studies, we here investigate not only the levels of VAR2CSA DBL-domain specific antibodies in sera from pregnant Malawian women but also further scrutinize the elicited antibody responses by exploring affinity of antibodies targeting VAR2CSA DBL5ε.

## Results

### Patient Characteristics

Pregnant women attending the Queen Elizabeth Central Hospital, Blantyre, Malawi in late third trimester of pregnancy were enrolled into a study of interactions between HIV and malaria in pregnancy, as described elsewhere [26], [31]. A convenience selection of serum samples collected on enrollment was used in the various assays of the present study, including a total of 189 serum samples from primigravidae, 21 from secundigravidae and 72 from multigravidae. HIV infection rates were lower than expected in the primigravidae group compared with the secundigravidae and multigravidae (χ^2^ test, p = 0.02316), which is probably a function of age and repeated exposures. Parasitemia was more common in primigravid women and much less common in multigravid women (χ^2^ test, p = 0.01594), consistent with the hypothesis that previous malaria infection during pregnancy produces VAR2CSA (and thus protective) antibodies. Maternal anemia rates and infant birth weights were not different among the groups (χ^2^ test, p>0.8). Patient characteristics are displayed in Table 1.

**Table 1:** Patient characteristics.

		Gravidity	
Variable	Primigravidae	Secundigravidae	Multigravidae
	(n = 189)	(n = 21)	(n = 72)
HIV infection	83 (43.9%)	13 (61.9%)	44 (61.1%)
Parasitemia	38 (20.3%)	3 (14.3%)	4 (5.6%)
Maternal anemia^a^	75 (39.7%)	11 (52.4%)	30 (41.7%)
Birth Weight ± SD, g	2942±404	2920±606	2933±552

a Anemia defined as hemoglobin levels <11 g/dl.

### VAR2CSA DBL-Domains Are Recognized by Immune Sera from Pregnant Adults

**Figure 1:**
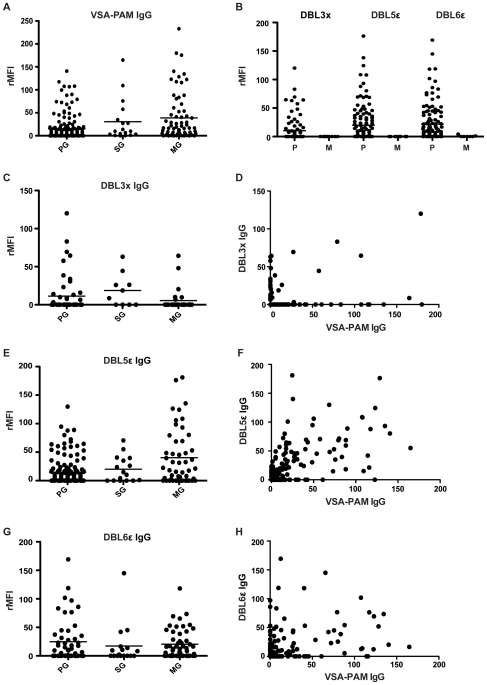
VSA-PAM and VAR2CSA DBL-domain recognition of naturally acquired antibodies in pregnant women. A: IgG levels against VSA-PAM expressed on the surface of CS2 parasites. Groups are divided into primigravidae (PG), secundigravidae (SG) and multigravidae (MG) (x-axis) and antibody levels are expressed as relative median fluorescence intensity (rMFI, y-axis). MG women have significantly higher levels of antibodies against VSA-PAM than PG women (Kruskal-Wallis ANOVA, p = 0.0001; Dunn's Multiple Comparison test, p<0.001). B: IgG levels against VAR2CSA domains DBL3x (n = 93 pregnancy and 10 male sera), DBL5ε (n = 125 pregnancy and 13 male sera), and DBL6ε (n = 122 pregnancy and 9 male sera), Groups are divided into pregnant women (all parities shown together, labeled P) and males (labeled M) from the same endemic areas. Pregnant women had significantly higher levels of antibodies against each domain than their male counterparts, except for DBL3x, probably due to the lower sample size for this domain (Kruskal-Wallis ANOVA, p<0.0001; Dunn's Multiple Comparison test, p>0.05 (ns) (DBL3x), p<0.01 (DBL5ε) and p = 0.05 (DBL6ε)). C: IgG levels against DBL3x showing no significant difference in antibody levels in PG and MG women (Kruskal-Wallis ANOVA, p = 0.0589). D: Correlation of IgG levels against DBL3x and total VSA-PAM, expressed as rMFI. No correlation was found (Spearman r = −0.08980, p = 0.3972). E: IgG levels against DBL5ε showing significantly higher levels of antibodies in MG women than PG women (Kruskal-Wallis ANOVA, p = 0.0141, Dunn's Multiple Comparison test, p<0.05). F: Correlation of IgG levels against DBL5ε and total VSA-PAM, expressed as rMFI. A significant positive moderate correlation was found (Spearman r = 0.5632, p<0.0001). G: IgG levels against DBL6ε showing no significant difference in antibody levels in PG and MG women (Kruskal-Wallis ANOVA, p = 0.5118). H: Correlation of IgG levels against DBL6ε and total VSA-PAM, expressed as rMFI. A significant positive weak correlation was found (Spearman r = 0.2905, p = 0.0013).

**Figure 2:**
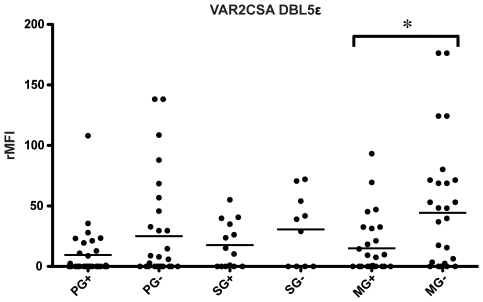
The effect of HIV infection on antibody levels to DBL5ε. IgG levels against DBL5ε showing HIV negative women to have higher antibody levels than HIV positive women in all parity groups, however only significantly so in multigravidae (Kruskal-Wallis ANOVA, p = 0.023). Groups are divided into primigravidae (PG), secundigravidae (SG) and multigravidae (MG) and HIV positive (+) or negative (−) (x-axis) and antibody levels are expressed as relative median fluorescence intensity (rMFI, y-axis).

We used flow cytometry to measure antibody levels in a panel of sera from Malawian pregnant women to the variant surface antigens (VSA) of *P. falciparum* VAR2CSA expressing lab isolate CS2 [32] (figure 1A and figure S1). This analysis showed multigravid (MG) women to have significantly higher levels of VSA-PAM antibodies than primigravid (PG) women from the same endemic area (Kruskal-Wallis ANOVA, p = 0.0001 followed by Dunn's Multiple Comparison test, p<0.001). We then used CHO-745 cells transfected with 3D7 VAR2CSA DBLdomains, described elsewhere [14], to investigate the domain specificity of these acquired antibodies (figure S2 and S3). Previous studies showed the various domains to be expressed on the surface of the transfected CHO-745 cells at similar magnitudes [19], and the level of surface expression was also monitored for each experiment in this study (figure S3). Using the panel of sera from Malawian pregnant women described above, we assayed levels of DBL domain specific antibodies. Flow cytometry assays using these VAR2CSA DBL domain transfected cells showed various patterns. After initial screening of all six DBL domains using serum samples from Malawian women with different parities (data not shown), additional serum samples were tested using domains DBL3x, DBL5ε and DBL6ε (Figure 1B). The reason for choosing these domains is the distinctly higher recognition of these domains by pooled hyperimmune multigravid sera than pooled male immune sera from individuals living in the same area, demonstrating a gender specific antibody recognition pattern of these three domains. The pattern of DBL3x recognition indicated that antibodies in sera from primigravidae show similar recognition of this domain to those in sera from multigravidae (Kruskal-Wallis ANOVA, p = 0.0589, figure 1C). Neither did antibody levels to DBL3x correlate with antibody levels to VSA-PAM antibodies (Spearman r = −0.08980, p = 0.3972, figure 1D). Notably, A4 DBL3x transfected CHO-745 cells were used as a complement to 3D7 DBL3x transfected CHO-745 cells since the 3D7 variant was shown to contain a 12-mer residue deletion and did not bind CSA [33]. From this further analysis, it is apparent that DBL5ε was the only domain to induce a clear parity dependent recognition pattern, with multigravid women boasting notably higher levels of anti-DBL5ε antibodies than primigravidae (Kruskall-Wallis ANOVA, p = 0.0141; Dunn's Multiple Comparison test, p<0.05 (PG versus MG), figure 1E). Levels of VSA-PAM antibodies and DBL5ε specific antibodies showed a significant positive moderate correlation (Spearman r = 0.5632, p<0.0001), suggesting that DBL5ε may be important in the acquired immune response against PAM (figure 1F). The pattern of VAR2CSA DBL6ε antibody recognition indicates this domain to also be recognized by multigravid women to a similar extent as primigravidae (Kruskall-Wallis ANOVA, p = 0.5118, figure 1G). Levels of antibodies against DBL6ε did show a significant positive weak correlation with levels of antibodies against VSA-PAM (Spearman r = 0.2905, p = 0.0013, figure 1H). Analyzing the effect of HIV infection on antibody levels to DBL5ε revealed HIV negative women to have higher antibody levels than HIV positive women in all parity groups, but the difference was only significant in multigravidae (Kruskall-Wallis ANOVA, p = 0.023, Dunn's Multiple Comparison test, p<0.05 (PG versus MG), figure 2). We also investigated the possible correlation between parasitemia (parasites per µl) at the time of serum collection and antibody levels against VSA-PAM as well as against DBL5ε (figure S4). We found a weak significant correlation (Spearman r = 0.2134, p = 0.0005) between antibodies against VSA-PAM and parasitemia, something that holds true also for primigravidae (Spearman r = 0.2819, p = 0.0002) and multigravidae (Spearman r = 0.2833, p = 0.0175), but not for secundigravide. We also found a weak significant correlation (Spearman r = 0.2000, p = 0.0123) between antibodies against VAR2CSA DBL5ε and parasitemia in primigraviade but nor for secundi- or multigravidae nor for these three groups together.

### Antibody Affinity against VAR2CSA DBL5ε

**Figure 3:**
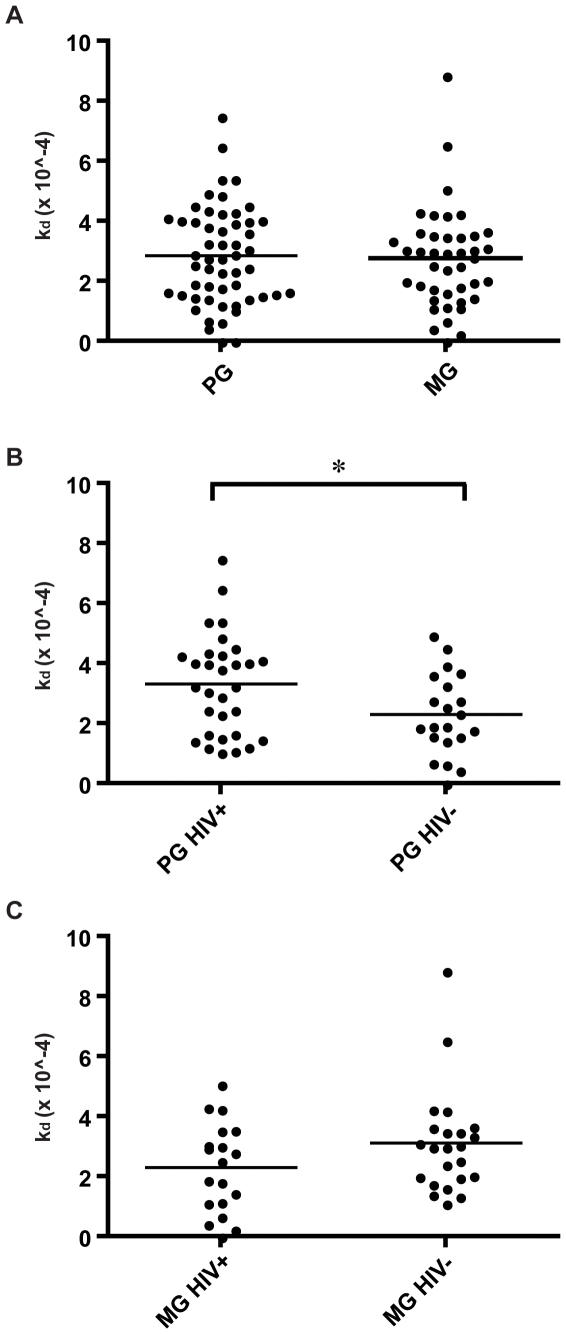
Affinity of naturally acquired antibodies in pregnant women to VAR2CSA DBL5ε. A: Affinity displayed as the dissociation rate constant (k_d_×10∧-4), comparing PG and MG (both HIV positive (+) and HIV negative (−) women). There is no significant difference between antibody affinities to DBL5ε comparing PG and MG women (t-test, p = 0.7630). B: Affinity displayed as the dissociation rate constant (k_d_×10∧-4), comparing primigravid women divided into HIV status. Antibodies from PG HIV- women have a significantly higher affinity to DBL5ε than antibodies from PG HIV+ women (t-test, p = 0.0230). C: Affinity displayed as the dissociation rate constant (k_d_×10∧-4), comparing multigravid women divided into HIV status. No significant difference in antibody affinity is seen comparing groups MG- women and MG+ women (t-test, p = 0.1152).

**Figure 4:**
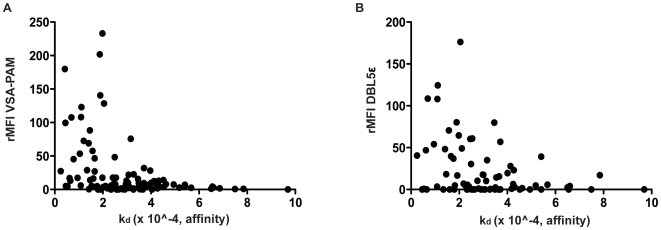
Correlation of VSA-PAM IgG, DBL5ε IgG and antibody affinity. A: Correlation of IgG levels against total VSA-PAM and antibody affinity against DBL5ε, expressed as rMFI and dissociation rate constant respectively. These show a negative moderate significant correlation (Spearman r = −0.3571, p = 0.0002). B: Correlation of IgG levels against DBL5ε and antibody affinity against DBL5ε, expressed as rMFI and dissociation rate constant respectively. These show a negative moderate significant correlation (Spearman r = −0.3414, p = 0.0024).

Due to the interesting pattern of VAR2CSA DBL5ε recognition by IgG in immune sera, we chose to continue working with this domain, investigating affinity properties of DBL5ε specific antibodies. Using recombinant FCR3 VAR2CSA DBL5ε and a surface plasmon resonance technique, we investigated the dissociation rate constant (k_d_) of immune antibodies as an indicator of their affinity. This technique allows comparison of different sera even though the concentration of the specific antibodies is not known, since k_d_ is concentration independent. Even though multigravid women have higher levels of antibodies against DBL5ε than primigravidae, these antibodies did not show higher affinity against this VAR2CSA DBL5ε domain (figure 3A). However, sera from primigravid HIV positive women showed significantly higher k_d_, hence lower affinity, than sera from primigravid HIV negative women, indicating HIV infection to impair the affinity of VAR2CSA DBL5ε domain specific antibodies (t-test, p = 0.0230, figure 3B). Among multigravid women, even though HIV negative women had higher levels of antibodies than HIV positive women, there was no significant difference in the affinity of these antibodies against DBL5ε (figure 3C). Malaria naïve sera showed binding only at background levels and as an extra control, we also used non-immune IgG and IgM as well as IgG depleted non-immune sera, neither exhibiting binding using the surface plasmon resonance technique (data not shown). Both antibody levels to total VSA-PAM and to DBL5ε correlated significantly with the dissociation rate constant (Spearman r = −0.3571, p = 0.0002 and r = −0.3414, p = 0.0024 respectively, figure 4A and B).

## Discussion

In this study investigating VAR2CSA DBL-domain specific immune response, we show the VAR2CSA domains DBL5ε and DBL6ε to be recognized in a gender specific manner (figure 1B). DBL5ε also displays a parity dependent recognition, as previously described for VSA-PAM antibodies [34], [35] suggesting this domain to be important in a protective response against PAM (figure 1E). All DBL-domains contain both conserved and polymorphic regions targeted by surface reactive antibodies, but the conserved regions are most prominent in DBL3x and DBL5ε [36]. Further, DBL5ε is highly conserved between both laboratory and clinical isolates, and most sequence differences localize to flexible loops in the protein [37], [38]. A recent study indicates that antibodies raised against DBL3x and DBL5ε domains are highly cross-reactive with several placental isolates [39]. We demonstrate a correlation between antibodies against a VAR2CSA expressing parasite line and DBL5ε domain specific antibodies, suggesting this domain to be a prominent target of antibodies developed in malaria infection during pregnancy. A study by Oleinikov *et al* showed that most antibodies elicited following immunization with DBL5ε and DBL6ε recognize polymorphic epitopes in the native protein [40]. Levels of adhesion-inhibitory antibody correlated with levels of anti-DBL5ε antibodies [41], and a recent study illustrated how antibodies raised against DBL3x and DBL5ε were highly cross-reactive with heterologous parasites [42]. Furthermore, in this study we used DBL5ε from three different parasite isolates (CS2, to measure total VSA-PAM antibody levels; 3D7 domains expressed in CHO cells; and FCR3 in affinity studies) with results correlating significantly (figure 4A and B). Also, the rMFI for antibodies against VSA-PAM and antibodies against DBL5ε have a very strong resemblance to each other across the population (figure 4A and B). Levels of anti-DBL5ε IgG have also been positively associated with birth weight, again indicating their protective effect [6]. A VAR2CSA vaccine will need to elicit a cross-reactive antibody response in order to be widely effective, and our data in conjunction with earlier studies [36], [41], [43] indicate DBL5ε to be a promising vaccine candidate.

In order to succeed in blocking iRBC adhesion to CSA in the placenta of pregnant women, it is possible that antibodies need not only to bind, but also to do so at strong affinity. Using the surface plasmon resonance technique, we found that even though multigravid women have slightly higher levels of antibodies against DBL5ε than primigravidae, there was no significant difference in the dissociation rate constant of antibodies against this VAR2CSA domain between the groups. Diving deeper into the different parities and the effect of HIV co-infection, antibodies against DBL5ε in primigravid HIV positive women tested had lower affinity than sera from HIV negative primigravidae from the same region (figure 3B), suggesting HIV infection to impair the strength of antibody binding. Among multigravid HIV, levels of DBL5ε antibodies were lower in HIV positive women (figure 2) but affinity was similar to that of antibodies present in HIV negative women (figure 3C). However, levels of total VSA-PAM antibodies as well as anti-DBL5ε antibodies correlate significantly with the dissociation rate constant used as an indicator of affinity of these antibodies (figure 4A and B). Since HIV in multigravid women seems to impair a pregnant woman's ability to control parasitemia, levels of antibodies are an important determinant of favorable birth outcomes and as a sign for less disease in these women [28]. Our data indicate that HIV infection impairs antibody affinity in primigravidae more than in multigravidae. One hypothesis would argue that antibody affinity is important in primigravidae when antibody levels are low whereas antibody levels and functionalities such as facilitation of opsonic activity may be more important determinants of outcome in multigravid women. Phagocytic activity of cytophilic antibodies is known to be impaired in multigravidae infected with HIV whereas no difference is seen in HIV infected and HIV uninfected primigravid women [23], [24].

Many studies have investigated presence, levels and function of antibodies targeting VSA-PAM, but less is known concerning the dynamics of binding interactions. It could be so that antibodies, to be protective, need to bind with high affinity. Some studies found no association between high levels of VSA-PAM antibodies and adhesion inhibition [44], whereas some clearly show this correlation [34]. We hypothesize that high affinity antibodies are protective whereas low affinity antibodies are less so and thereby rely more on increased levels and various antibody functions independent of the antibody binding equilibrium such as phagocytic activity and ability to block placental adhesion.

When designing a vaccine against pregnancy-associated malaria, various properties of antibody response against VSA-PAM are important to consider, especially in the presence of HIV co-infection. Conserved surface epitopes are interesting as possible vaccine components, if immune sera from several parts of the world recognize these epitopes. Considering that the DBL5ε specific response correlates well with acquired antibodies to VSA-PAM expressed on iRBC surface selected for VAR2CSA expression, this is an interesting domain for vaccine development. This study presents important information on altered mechanisms of antibody response toward VSA-PAM in presence of HIV infection, something that is of great interest in the continued quest for an efficient vaccine against pregnancy-associated malaria.

## Materials and Methods

### Ethics Statement

The study was approved by the College of Medicine Research and Ethics Committee, University of Malawi, the Royal Melbourne Hospital Clinical Research Ethics Committee, and the Institutional Review Boards of the Universities of Michigan and North Carolina. Written informed consent was sought from all eligible women involved in the study for blood collection and HIV testing. Adult males gave written informed consent for collection of blood as part of studies approved by the same committee.

### VAR2CSA Transfectants

The six domains of 3D7 PFL0030c *var2csa* (Genbank accession no. NP_701371) and DBL3x of A4 PFL0030c *var2csa* (Genbank accession no. AAQ73926) were amplified from genomic DNA by PCR and cloned into the pSRα5-12CA5 vector (Affymax Research Institute, Palo Alto, CA) as described [14]. Chinese hamster ovary PgsA-745 (CHO-745) cells deficient in glycosaminoglycans (American Type Culture Collection, Manassas, VA) were transfected with these plasmids, and stable transfectants expressing the various DBL-domains were used for flow cytometry assays.

### Measurements of VSA-PAM Antibodies by Flow Cytometry

IRBCs of *P. falciparum* laboratory strain CS2 at 5–10% parasitemia of trophozoites were washed three times in PBS containing 1% fetal calf serum (PBS-1% FCS). Cells at 0.1% hematocrit (100 µl) were incubated with test sera at a 1∶20 dilution in PBS-1% FCS for 30 min in 96-well v-bottom plates. This was followed by a 30-min incubation with 50 µl rabbit anti-human IgG (DAKO A0424) at a 1∶100 dilution in PBS-1% FCS and a final incubation with 50 µl Alexa Fluor 488-conjugated donkey anti-rabbit IgG (Molecular Probes A-21206) at a 1∶500 dilution and 10 mg/ml ethidium bromide at a 1∶100 dilution in the dark for 30 min. All incubations were performed at room temperature and after each incubation, the cells were washed three times in PBS-1% FCS. After the final wash, cells were resuspended in 200 µl PBS and analyzed with a FACSCalibur flow cytometer with Cell Quest software (BD Biosciences). Included in all runs were a positive control made up of a pool of hyperimmune sera from pregnant women resident in Blantyre, Malawi, and two negative controls made up of pooled sera from malaria non-exposed adults in Melbourne, Australia. The geometric mean fluorescence intensity (MFI) was calculated as a measure of IgG binding to iRBC. IRBCs were gated and 1000 positive cells were collected on the basis of ethidium bromide fluorescence. Samples were determined to have antibodies if the MFI was greater than the mean of the negative controls plus 2 standard deviations (SD). Sample readings were assigned relative values by using the formula ((sample MFI) – (negative control MFI))/((positive control MFI) – (negative control MFI))*100.

### Measurements of VAR2CSA DBL-Domain Specific Antibodies by Flow Cytometry

Transfectant CHO-cells were washed in PBS and pre-blocked in 2% BSA containing 10% FCS for 45 min at room temperature (RT). In each reaction, 2×10^5^ cells (200 µl of cell suspension) were added to each well in 96-well v-bottom plates. Transfectants were then incubated with individual serum samples at a 1∶200 dilution for 45 min before addition of polyclonal rabbit anti-human IgG (DAKO) at a 1∶100 dilution for 45 min. Subsequently, cells were incubated with Alexa-Fluor 488 donkey anti-rabbit IgG (Molecular Probes), 1∶500 dilution for 45 min in dark. Sera and antibodies were diluted in PBS containing 2% FCS. Between each step, cells were washed three times with PBS- 2% FCS. After the final three washes, cells from each reaction were resuspended in 200 µl PBS, moved to FACS tubes and directly analyzed on a FACSCalibur flow cytometer (BD Biosciences). All samples were run in duplicates and at least 10 000 viable cells were analysed per sample. The domain expression level of each tranfectant was monitored during each run by using a monoclonal mouse-hemagglutinin antibody (1∶200, Roche) followed by Alexa-Fluor 488 donkey anti-mouse IgG (Molecular Probes). Pooled serum samples from Malawian hyperimmune multigravid women were used as a positive control, and pooled serum samples from malaria-naïve Melbourne donors were used as a negative control. The CellQuest software (BD Biosciences) were used to determine geometric mean fluorescence intensity (MFI), and relative MFI (rMFI) was determined by formula:

((sample MFI) – (negative control MFI))/((positive control MFI) – (negative control MFI))*100

### Biacore Surface Plasmon Resonance

Antibody affinity to VAR2CSA DBL5ε measurements was performed on a BIAcore 3000 instrument (Biacore AB, Uppsala Sweden). Recombinant FCR3 VAR2CSA DBL5ε and the control 3D7 DBL6γ (kind gift from Ali Salanti) was coupled to a CM5 sensor chip (Biacore AB) using an amine coupling kit, according to the instructions of the manufacturer. FCR3 VAR2CSA DBL5ε in sodium acetate pH 4.5 and 3D7 DBL6γ in sodium acetate pH 4.8 were injected to flow cell 2 and 3 (FC2 and 3) respectively to be immobilized on the sensor surface reaching a total of 1200 resonance units (RU). No protein was injected into FC1, in order to serve as control for background binding to the dextran matrix. A continuous flow of HBS-EP buffer (0.01 M Hepes, pH 7.4, 0.15 M NaCl, 3 mM EDTA and 0.005% (v/v) Surfactant P20, Biacore AB) passing over the sensor surface at 30 µl/min was maintained and experiments were performed at 25°C. Each serum sample to be tested was diluted 1∶7.5 and 1∶15 in HBS-EP buffer. Pooled serum samples from Malawian hyperimmune multigravid women were used as a positive control. Sera from malaria-naïve Melbourne donors, non-immune IgG and IgM as well as sera depleted from IgG were used as controls. The sensor chip was regenerated with a pulse of 10 mM glycine pH 1.5. The dissociation rate constant, k_d_, was evaluated for each sample showing specific DBL5ε binding, using the software BIAevaluation 3.0 (Biacore AB).

### Statistical Analysis

Statistical analyses were performed using Excel and GraphPad Prism and InStat3. Appropriate tests were performed (Kruskal-Wallis ANOVA followed by Dunn's Multiple Comparison Test (figure 1 and 2), Spearman rank test (figure 4, figure S4) for the non-normal distributed data) and t-test for the normal distributed data (figure 3)) depending on properties of respective data.

## Supporting Information

Figure S1 Flow cytometry plots of VSA-PAM antibody measurements. using iRBC. A: Settings for detection of RBC. FSC voltage, amplifier gain 2.00. SSC voltage 352, amplifier gain 1.00. B: To define iRBC (stained with ethidium bromide) and uninfected RBC (uRBC), gates according to fluorescent channel was created. uRBC population show negative readings while iRBC have high MFI readings.
(0.63 MB TIF)

Figure S2 Flow cytometry plots of CHO-cells. A. FSC/SSC plot showing our gated population of non-transfected CHO cells. Same gate was used for all experimental analysis. B. FSC/SSC plot showing our gated population of DBL5ε transfected CHO cells. Same settings were used for all non-transfected and all transfected cells.
(0.52 MB TIF)

Figure S3 Overlays of histograms with transfected. and non-transfected CHO cells. A–C. Overlays of histograms showing fluorescence intensity of CHO cells transfected with VAR2CSA DBL3x, DBL5ε and DBL6ε (gray peaks) respectively, and non-transfected CHO cells (black peaks). Both were incubated with a pool of sera from multigravid women (used as a positive control) and a secondary antibody labelled with Alexa-488 (FL1). The y-axis shows the normalized peak height and the x-axis show the fluorescent intensity in fluorescent channel 1 (FL1-H). D. Histogram overlay of CHO cells transfected with VAR2SA DBL3x (red line), DBL5ε (gray) and DBL6ε (blue line) respectively, and non-transfected CHO cells (black). All cells were incubated with an anti-hemagglutinin (HA) antibody (targeting the transfection construct) followed by a secondary antibody labelled with Alexa-488 (FL1). Transfection levels of these three stable transfectants were consistently great and highly similar to one another.
(0.91 MB TIF)

Figure S4 Correlation between antibody levels and parasitemia. Correlation between parasitemia (parasites per µl) at the time of serum collection and antibody levels against VSA-PAM as well as against DBL5ε. We found a weak significant correlation (Spearman r = 0.2134, p = 0.0005) between antibodies against VSA-PAM and parasitemia (A), something that holds true also for primigravidae (Spearman r = 0.2819, p = 0.0002) (C) and multigravidae (Spearman r = 0.2833, p = 0.0175) (G), but not for secundigravide (E). We also found a weak significant correlation (Spearman r = 0.2000, p = 0.0123) between antibodies against VAR2CSA DBL5ε and parasitemia in primigravidae (D) but nor for secundi- or multigravidae (F and H) nor for these three groups together (B).
(0.94 MB TIF)
