# Supplementary figures and images for: Differential Recognition of P. falciparum VAR2CSA Domains by Naturally Acquired Antibodies in Pregnant Women from a Malaria Endemic Area
Source: PLoS One. 2010 Feb 16;5(2):e9230. doi: 10.1371/journal.pone.0009230 (PMC2821912; doi:10.1371/journal.pone.0009230)

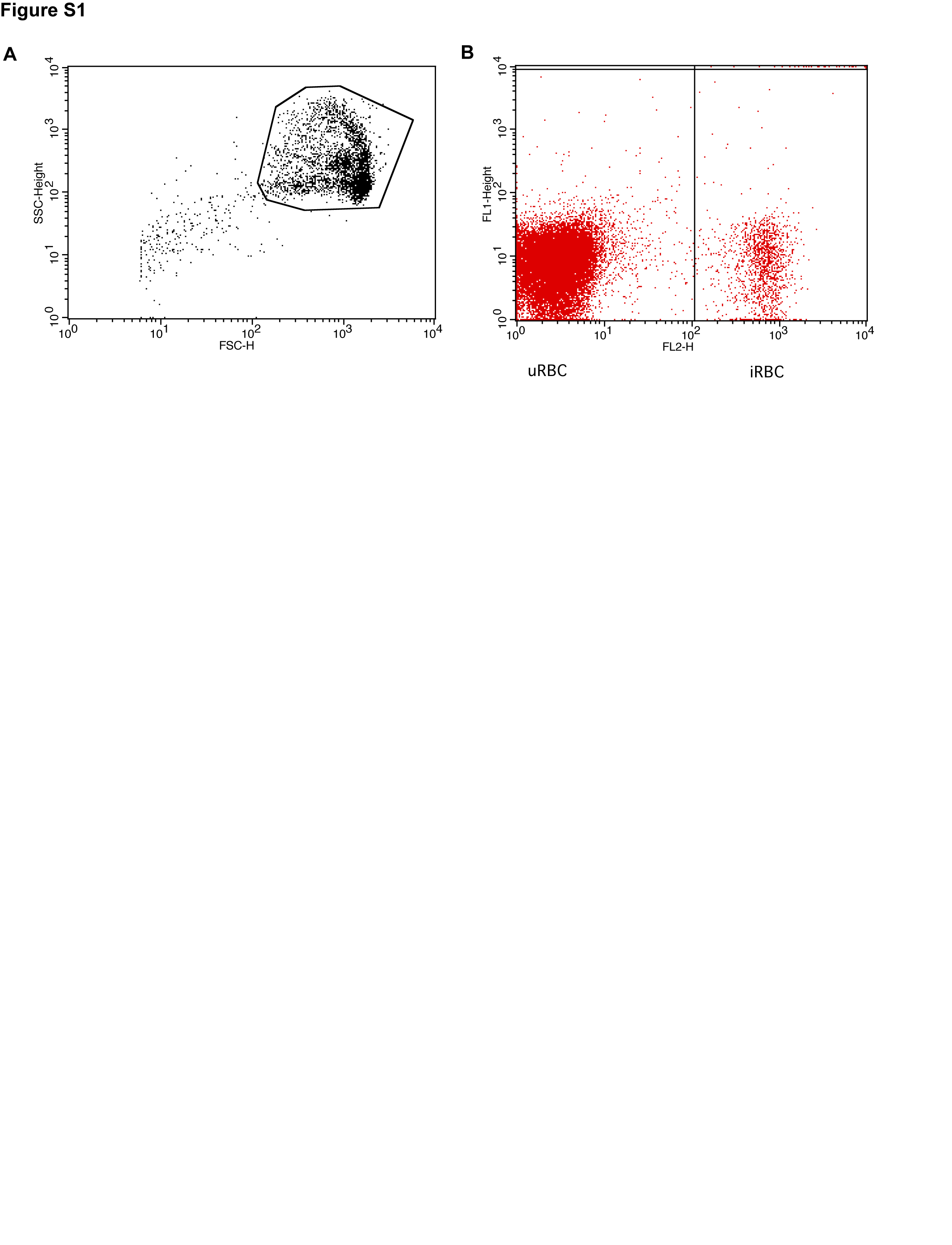

Supplement: Figure S1 — Flow cytometry plots of VSA-PAM antibody measurements. using iRBC. A: Settings for detection of RBC. FSC voltage, amplifier gain 2.00. SSC voltage 352, amplifier gain 1.00. B: To define iRBC (stained with ethidium bromide) and uninfected RBC (uRBC), gates according to fluorescent channel was created. uRBC population show negative readings while iRBC have high MFI readings. (0.63 MB TIF) [file pone.0009230.s001.tif]

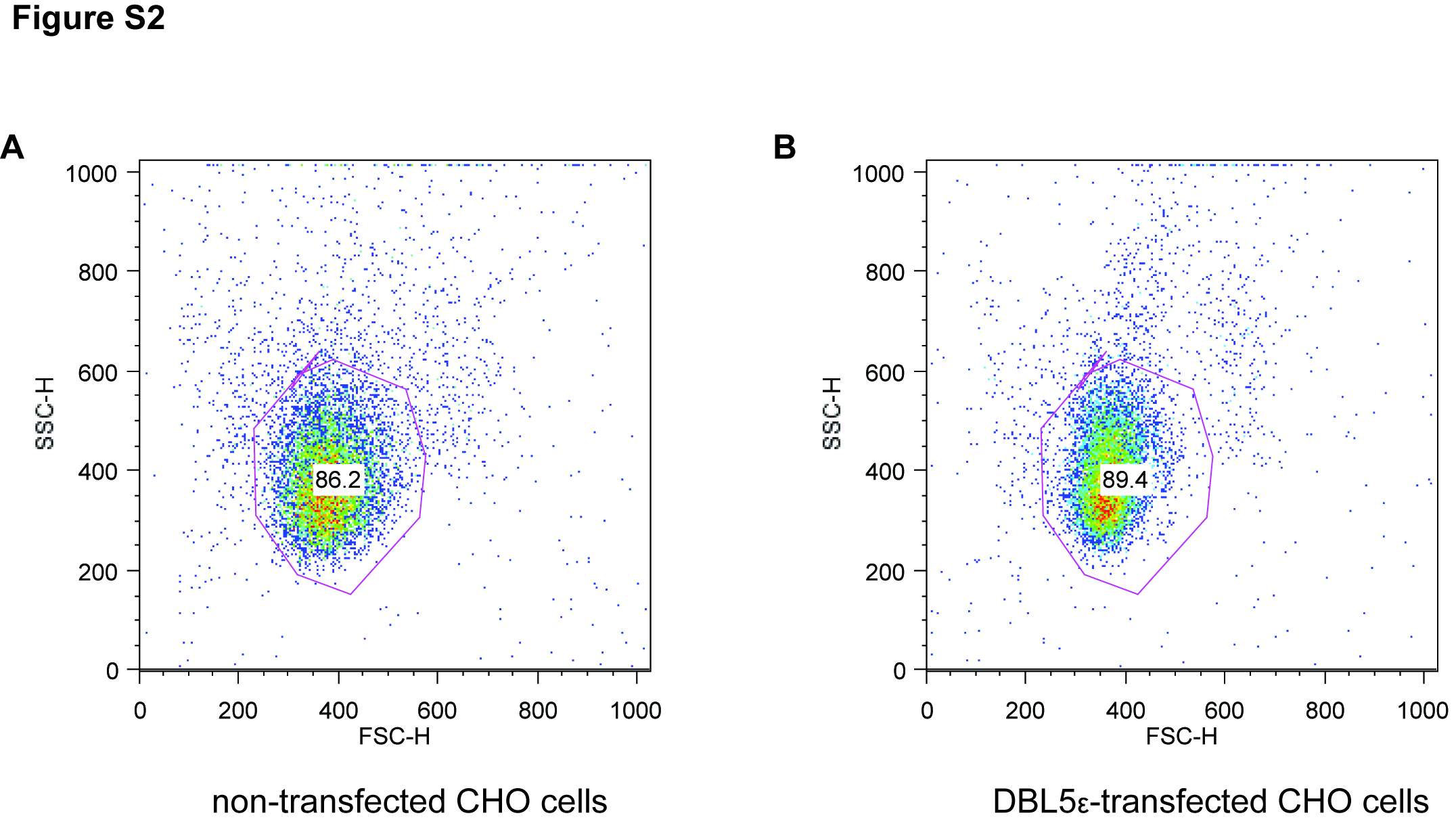

Supplement: Figure S2 — Flow cytometry plots of CHO-cells. A. FSC/SSC plot showing our gated population of non-transfected CHO cells. Same gate was used for all experimental analysis. B. FSC/SSC plot showing our gated population of DBL5ε transfected CHO cells. Same settings were used for all non-transfected and all transfected cells. (0.52 MB TIF) [file pone.0009230.s002.tif]

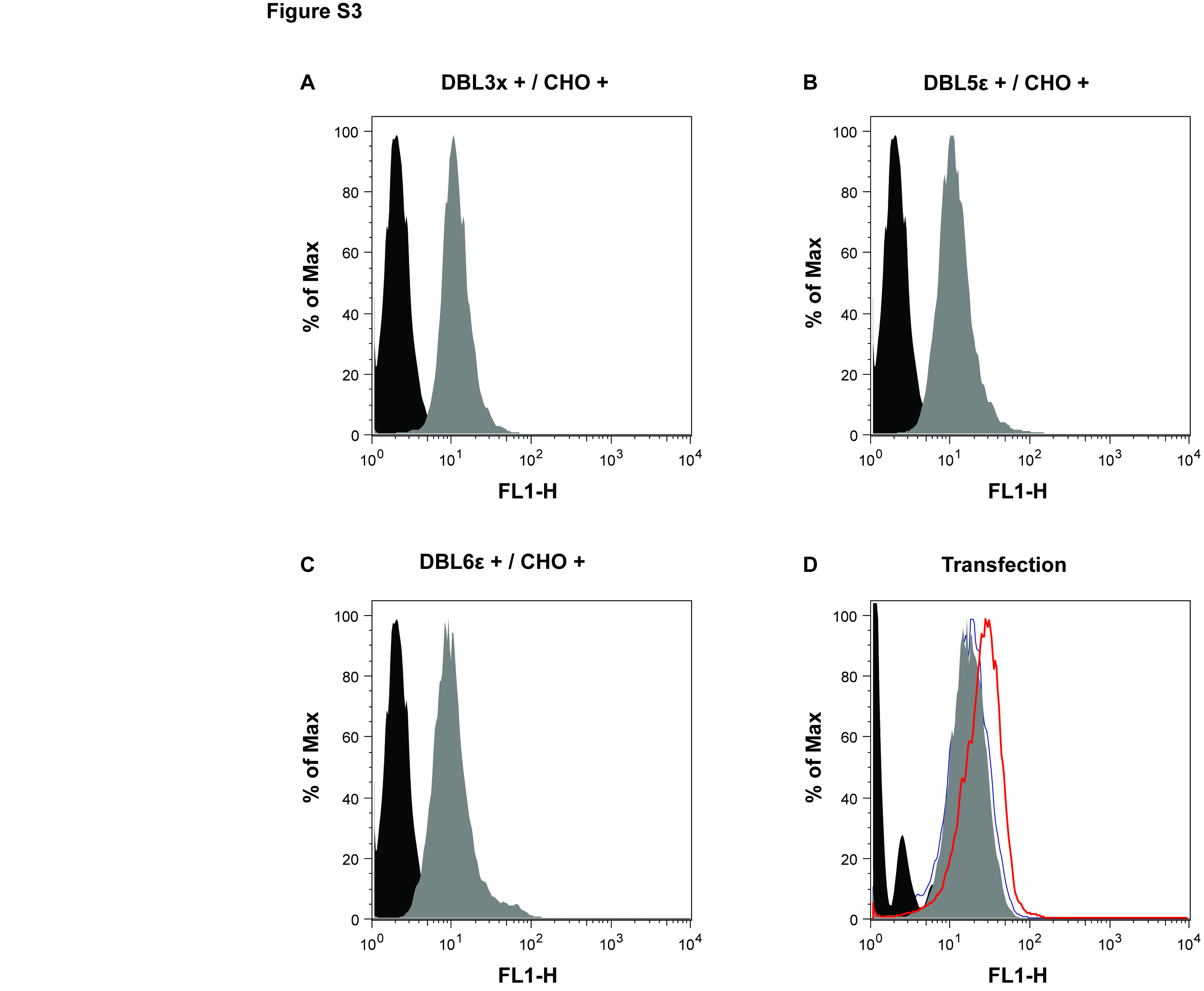

Supplement: Figure S3 — Overlays of histograms with transfected. and non-transfected CHO cells. A–C. Overlays of histograms showing fluorescence intensity of CHO cells transfected with VAR2CSA DBL3x, DBL5ε and DBL6ε (gray peaks) respectively, and non-transfected CHO cells (black peaks). Both were incubated with a pool of sera from multigravid women (used as a positive control) and a secondary antibody labelled with Alexa-488 (FL1). The y-axis shows the normalized peak height and the x-axis show the fluorescent intensity in fluorescent channel 1 (FL1-H). D. Histogram overlay of CHO cells transfected with VAR2SA DBL3x (red line), DBL5ε (gray) and DBL6ε (blue line) respectively, and non-transfected CHO cells (black). All cells were incubated with an anti-hemagglutinin (HA) antibody (targeting the transfection construct) followed by a secondary antibody labelled with Alexa-488 (FL1). Transfection levels of these three stable transfectants were consistently great and highly similar to one another. (0.91 MB TIF) [file pone.0009230.s003.tif]

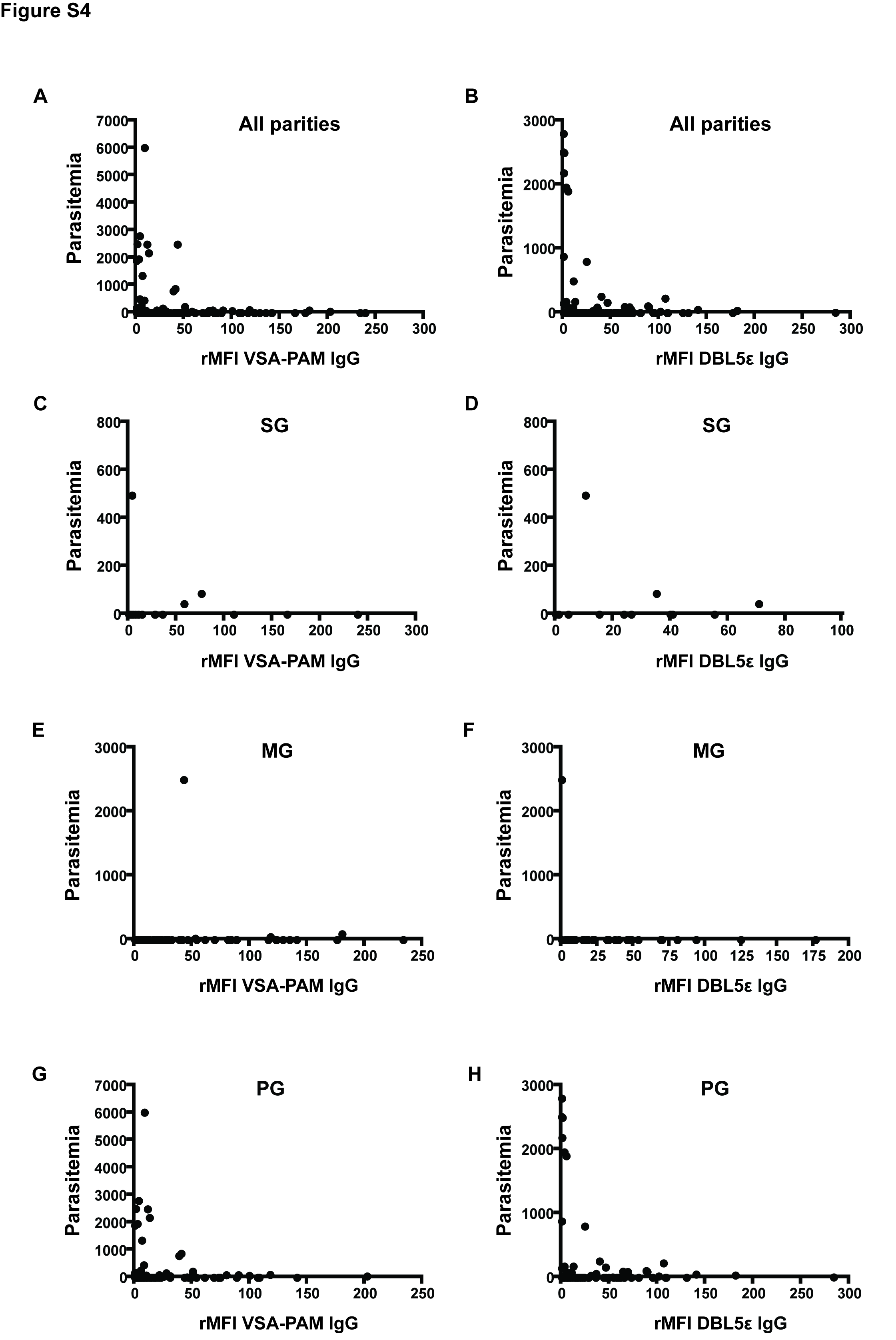

Supplement: Figure S4 — Correlation between antibody levels and parasitemia. Correlation between parasitemia (parasites per µl) at the time of serum collection and antibody levels against VSA-PAM as well as against DBL5ε. We found a weak significant correlation (Spearman r = 0.2134, p = 0.0005) between antibodies against VSA-PAM and parasitemia (A), something that holds true also for primigravidae (Spearman r = 0.2819, p = 0.0002) (C) and multigravidae (Spearman r = 0.2833, p = 0.0175) (G), but not for secundigravide (E). We also found a weak significant correlation (Spearman r = 0.2000, p = 0.0123) between antibodies against VAR2CSA DBL5ε and parasitemia in primigravidae (D) but nor for secundi- or multigravidae (F and H) nor for these three groups together (B). (0.94 MB TIF) [file pone.0009230.s004.tif]
